# Modeling of a Rope-Driven Piezoelectric Vibration Energy Harvester for Low-Frequency and Wideband Energy Harvesting

**DOI:** 10.3390/mi12030305

**Published:** 2021-03-15

**Authors:** Jinhui Zhang, Maoyu Lin, Wei Zhou, Tao Luo, Lifeng Qin

**Affiliations:** Department of Mechanical and Electrical Engineering, Xiamen University, Xiamen 361005, China; jinhuizhang@xmu.edu.cn (J.Z.); mylin@stu.xmu.edu.cn (M.L.); weizhou@xmu.edu.cn (W.Z.); luotao@xmu.edu.cn (T.L.)

**Keywords:** piezoelectric vibration energy harvester, low frequency, wideband, modeling

## Abstract

In this work, a mechanical model of a rope-driven piezoelectric vibration energy harvester (PVEH) for low-frequency and wideband energy harvesting was presented. The rope-driven PVEH consisting of one low-frequency driving beam (LFDB) and one high-frequency generating beam (HFGB) connected with a rope was modeled as two mass-spring-damper suspension systems and a massless spring, which can be used to predict the dynamic motion of the LFDB and HFGB. Using this model, the effects of multiple parameters including excitation acceleration, rope margin and rope stiffness in the performance of the PVEH have been investigated systematically by numerical simulation and experiments. The results show a reasonable agreement between the simulation and experimental study, which demonstrates the validity of the proposed model of rope-driven PVEH. It was also found that the performance of the PVEH can be adjusted conveniently by only changing rope margin or stiffness. The dynamic mechanical model of the rope-driven PVEH built in this paper can be used to the further device design or optimization.

## 1. Introduction

In recent years, the great demand for micro-energy harvesting devices has been continuously increasing with the wide application of wearable device and wireless sensors due to the limited life-span and disposal pollution of the battery [1,2,3]. Mechanical vibration energy has been one of the major energy sources due to its ubiquity in the environment. The mechanisms for vibration energy harvesting can be mainly categorized into electromagnetic [4,5,6], electrostatic [7,8,9], piezoelectric [10,11,12,13], and triboelectric [14,15,16,17] ways. Since piezoelectric mechanism has a characteristic of simple structure, high mechanical-electrical conversion, and compatibility with CMOS (Complementary Metal Oxide Semiconductor), PVEH has been a hot research area in the past two decades for making self-powered sources to power small-scale systems [18,19,20]. For example, Kuang et al. proposed a PVEH combining magnets was designed to wear on the leg and could scavenge energy from knee-joint motions during human walking to provide sustainable energy supply for body sensors to realize energy-autonomous wireless sensing systems [21].

A traditional PVEH is typically composed of a piezoelectric cantilever beam and a proof mass attached to the free end of the cantilever. These kind of PVEHs usually have a high resonant frequency, especially for the micro PVEHs whose resonant frequency can reach thousands of hertz (shown in Table 1). Hence, it is difficult for the traditional cantilever beam type PVEH to be applied in many practical environment where the vibration frequency is quite low (<100 Hz, shown in Table 2).

Meanwhile, traditional PVEHs are usually single-mode resonant systems with relatively narrow bandwidth. Power output will be significantly reduced when the resonant frequency of a resonator and the ambient vibration frequency is mismatched. Therefore, it is a big challenge for PVEH to be used for in most practical applications, where the vibration frequency is time-variable. To address this issue, Leland and Wright proposed one technique to tune the resonant frequency of the harvester from 200 to 250 Hz by applying an axial compressive load since the resonant frequency of cantilever beam is stress dependent [32]. Similar research on resonant frequency shift by preload was also studied by Eichhorn et al., showing that one harvester could be altered from 380 to 292 Hz for a compressive preload, and another harvester was tuned from 440 to 460 Hz by applying a tensile preload [33]. However, the frequency tunable performance of these harvesters was achieved manually with the aids of preload mechanical structure. Hence, enabling the harvesters to automatically adjust its resonance frequency to match the driving frequency is still a challenge. Another strategy is to develop wideband PVEH whose working frequency can cover the frequency range of the vibration source as much as possible.

For achieving wideband energy harvesting, various methods have been proposed [34,35,36], which can be mainly categorized into multimodal technique and nonlinear techniques. As for multimodal technique, it usually uses an array of beams with different resonant frequencies or designing one beam to have multiple vibrational modals [37,38,39]. For example, Wang et al. [37] and Liu et al. [38] demonstrated that the array-beams PVEH could achieve wider bandwidth if the number of beams were added, where each beam had different frequency characteristics. Wu et al. [40] presented a PVEH based on one M-shaped beam comprising a main beam and two dimension-varied folded auxiliary beams interconnected through a proof mass at the end of the main cantilever. Such an energy harvester owns a three degree-of-freedom vibrating mode, and the resonant frequencies of its first three orders can be tuned close enough to obtain a utility wide bandwidth. Compared with tradition single-mode resonator system, the bandwidth of harvester can be greatly improved by multimodal mechanism, and wider or even unlimited bandwidth could be theoretically achieved if the number of beams continuously add for array-beams harvester. However, the disadvantage of multimodal technique is low volume efficiency because the fact that the bandwidth of single beam is not broadened. 

On the contrary, the wideband energy harvesting using nonlinear techniques is increasing the bandwidth of single energy harvester based on nonlinear mechanism [35,41,42,43,44]. The most common nonlinear mechanism is the frequency up-conversion (FUC) technique, which can broaden the frequency bandwidth efficiently and offer a PVEH MEMS (Micro-Electro-Mechanical System) solution for low-frequency vibration energy harvesting. Generally, these frequency up-conversion (FUC) technologies can be classified into the contact and non-contact types. In terms of contact type, research has most commonly been conducted on the impact-driven PVEH that uses contact-mechanical force [11,43,45,46,47,48] to convert vibration energy to electrical energy. For example, Liu et al. [45] realized wideband energy harvesting and high output power via direct impact of a high-frequency piezoelectric Pb(Zr_x_Ti_1-x_)O_3_ (PZT) beam using a low-frequency driving beam. Vijayan et al. [49] investigated a modified design in which a spring element is attached to one end of a beam, which can avoid damage to the device due to the direct mechanical impact between the hard beams. As for the harvesters using the non-contact FUC technique, non-contact forces such as an extra magnetic force [50,51,52,53,54,55] are used to convert vibration energy to electrical energy. For example, Tang et al. [52] proposed a bi-stable FUC PVEH that consists of a central sliding mass and a pair of piezoelectric generators. Permanent magnets are mounted on both the central mass and the ends of the generators. The sliding mass moves back and forth under the ambient excitation and intermittently repels generators to oscillate by the mutually repulsive magnetic force. Since it is non-contact frequency up-conversion, thereby avoiding mechanical collision and improving long-term working durability. Izadgoshsb et al. [56] presented a PVEH consisting of a double-pendulum and a PZT cantilever beam with magnets on their ends. By coupling the rotatable magnetic force interactions between the ends of the pendulum and PZT cantilever beam, the double-pendulum can impact the PZT cantilever beam for multiple times within one excitation period. Hence, the performance of the double-pendulum-based system in harvesting energy from low frequency human motions can be effectively improved compared to the conventional PVEH and single pendulum-based system. Although the nonlinear technique has been proven to be very efficient on broadening the bandwidth of harvester, the resulted bandwidth is still limited.

**Figure 1:**
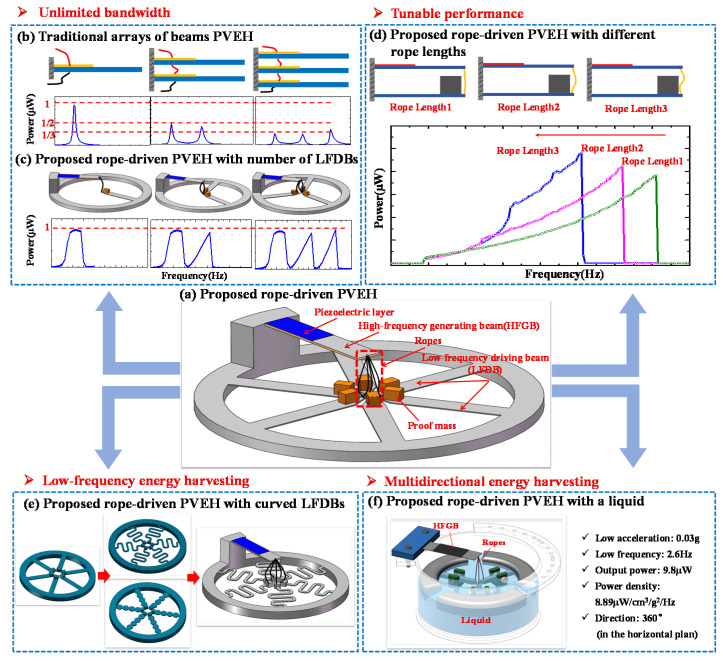
Schematic of the proposed novel low-frequency wideband piezoelectric energy harvester based on rope-driven mechanism for wideband, low-frequency, and multi-directional energy harvesting: (**a**) proposed rope-driven PVEH; (**b**) performance of traditional arrays of beams PVEH vs. number of beams; (**c**) performance of proposed rope-driven PVEH vs. number of LFDBs; (**d**) proposed rope-driven PVEH vs. rope lengths; (**e**) proposed rope-driven PVEH with curved LFDBs; (**f**) proposed rope-driven PVEH using liquid as energy capturing medium.

Hence, we proposed a low-frequency and wideband harvester based on a hybrid frequency up-conversion (FUC) nonlinear and multimodal mechanism in our previous work [57], aiming to combine the advantages of these two mechanisms, as demonstrated in Figure 1. The proposed wideband PVEH system (shown in Figure 1a) is composed of one high-frequency beam attached with piezoelectric material as the generating beam (HFGB) and multiple low-frequency beams with different frequencies as driving beams (LFDBs). Multiple LFDBs are connected mechanically with the HFGB using ropes. The main advantages of the rope-driven PVEH can be described as below:

(a) Wider or even unlimited bandwidth could be achieved if the number of LFDBs are continuously increased. Unlike wideband PVEH based on an array piezoelectric beams in serial/parallel connection, the output performance of proposed PVEH will not deteriorate with the changing number of LFDBs, which has theoretically and experimentally been proved, and Figure 1b,c show a typical experimental result [57].
(b) Similar to the impact-driven FUC wideband PVEH using a stopper, when an individual LFDB pulls the HFGB to oscillate it can achieve wideband energy harvesting, named as rope-driven FUC mechanism. Additionally, impact and rope-driven FUC mechanism can occur in the proposed PVEH by properly setting the length of rope, thus a much wider bandwidth could be achieved compared with the conventional impact-based FUC nonlinear wideband PVEH. Moreover, the working frequency of proposed PVEH can be tuned without re-fabricating or damaging the original structure by simply changing the rope length, which is ultra-convenient for practical applications. All these features of proposed PVEH has been experimentally verified, as shown in Figure 1d [58].
(c) Only HFGB is used for output in the proposed PVEH, which does not require a piezoelectric layer on LFDB, allowing great flexibility on the structure design of LFDB for various applications. For example, LFDB can adopted a curved shape shown in Figure 1e, which makes it easy to achieve low-frequency energy harvesting in a vibration environment, such as human motion, engine vibration, moving vehicles, and wave motion. Moreover, ultralow frequency, low intensity, and multidirectional vibration energy harvesting in a horizontal plane can be achieved if a liquid-based system is used as LFDB (see Figure 1f), which is difficult to be realized with traditional PVEHs [59].

The mainly characteristics of the proposed PVEH have been demonstrated and discussed in detail in our prior work [57,58]. Whereas the dynamic process of LFBD driving HFGB is complicated, and multiple parameters such as acceleration, rope margin and rope stiffness, will greatly affect performance of harvester, which has not been systematically studied. Hence, in this paper, a rope-driven PVEH consisted of one LFDB and one HFGB is presented as a basic structure to systematically investigate the effects of the parameters in the performance of the PVEH by theoretical modeling and experimental verification. Here, a mechanical dynamic model is established based on mass-spring-damper systems, which can be used to predict the dynamic motion of the LFDB and HFGB effectively using lumped parameter modeling approach. The effects of multiple parameters, including acceleration, rope margin and rope stiffness, have been clarified systematically by numerical simulation and experiments using this model. The results show a reasonable agreement between the simulation and experimental study. This work will provide a basic mechanism understanding and give guidance for the design of rope-driven PVEH. The rest of the paper is organized as follows: Section 2 gives a mechanical model of the basic structure of rope-driven PVEH. Section 3 describes the experiment and simulation procedure. Section 4 mainly shows the parameter study on PVEH based on simulation and experiments, and Section 5 presents the conclusions drawn from the simulation and experimental results.

## 2. Modeling

**Figure 2:**
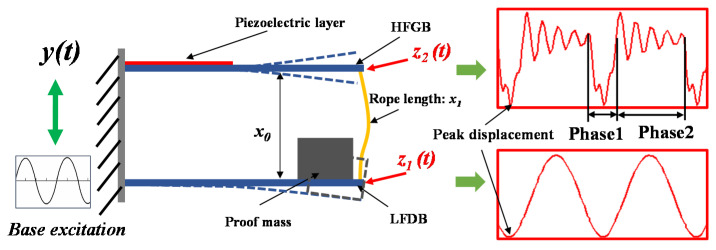
Schematic of the proposed PVEH.

The operation principle of the rope-driven PVEH with one LFDB and one HFGB is illustrated in Figure 2. The rope-driven PVEH is mounted on a base. Under a periodic excitation of the base, the LFDB pulls HFGB and transfers mechanical energy to HFGB by the rope for a short period in each vibration cycle when the LFDB is excited to exceed the margin *Δx* (defined as length of rope *x*_1_ minus initial distance of two beams *x*_0_). This stage is named as Phase 1 (rope-driven vibration). This pull will give rise to a retardation of the LFDB’s vibration amplitude but broaden the operating frequency bandwidth of LFDB. The reason is that HFGB arranged above the LFDB can be treated as a stopper just like the conventional impact-driven PVEH. As the rope pulls the HFGB, the effective stiffness of LFDB increases suddenly and results in a higher resonant frequency. Hence, the resonance of PVEH is extended over a wider interval of the frequency spectrum. After Phase 1, HFGB obtains vibrational energy from the LFDB and vibrates with exponentially attenuating amplitude at its higher resonant frequency (shown in Figure 2), and the cyclic deformation of piezoelectric material on HFGB will be transformed into electricity based on piezoelectric effect. LFDB continues to be excited by the base excitation until it pulls the HFGB in the next cycle. This phase is thought as Phase 2 (free vibration). The LFDB pulling the HFGB will not happen when the excitation is not large enough, and they will be forced to vibrate at the frequency of base excitation.

**Figure 3:**
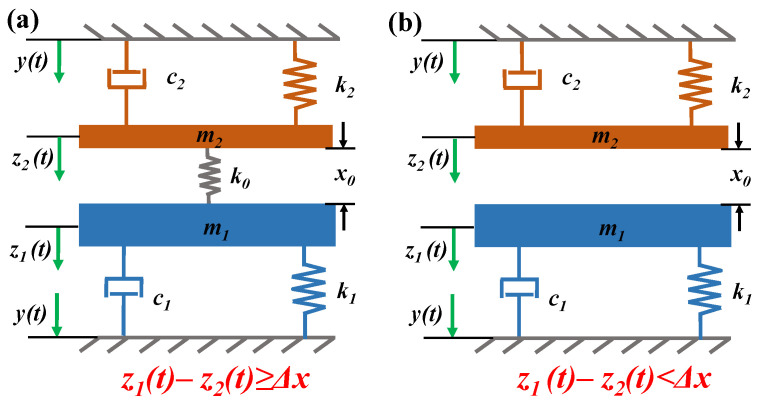
Mechanical model of the proposed PVEH as (**a**) *z*_1_ − *z*_2_ *≥ Δx* and (**b**) *z*_1_ − *z*_2_ *< Δx*.

A mechanical model of the rope-driven PVEH is established to describe the dynamic motion of PVEH base on the above mentioned working principle, as seen in Figure 3. Following the similar procedure [45], LFDB and HFGB can be modeled as two mass-spring-damper systems. LFDB, which is considered to be one suspension system, consists of a proof mass *m*_1_ suspended by a spring *k*_1_ and a damper *c*_1_. HFGB is another suspension system, acting as a generator. Initially, HFGB is placed at a distance of *x*_0_ above the LFDB, and has a damping coefficient *c*_2_, stiffness *k*_2_, and proof mass *m*_2_. The base excitation *y(t)* causes the proof mass *m*_1_, *m*_2_ to move relatively to the base movement (*y(t)*) as *z*_1_*(t)*, *z*_2_*(t)*, respectively. When the relative displacement of *z*_1_*(t)* − *z*_2_*(t)* is bigger than rope margin *Δx* (defined as length of rope *x*_1_ minus initial distance of two beams *x*_0_), the LFDB will pull HFGB by the rope. At this time, the rope can be treated as a massless spring with stiffness of *k*_0_ considering the mass of rope is much less than the *m*_1_, *m*_2_ as shown in Figure 3a. Otherwise, when *z*_1_*(t)* − *z*_2_*(t)* is smaller than *Δx*, the rope is ignored (see in Figure 3b). Hence, corresponding to the mechanical model, the equations for dynamic motion of LFDB and HFGB’s tip movement (*z_1_(t)*, *z_2_(t)*) can be built.

(1) $$LFDB:\;\left\{ \begin{array}{ll} m_{1}{\ddot{z}}_{1}+c_{1}{\dot{z}}_{1}+k_{1}z_{1}+k_{0}((z_{1}-z_{2})-\Delta x)=-m_{1}\ddot{y} & \left(z_{1}-z_{2}\geq \Delta x\right)\\ m_{1}{\ddot{z}}_{1}+c_{1}{\dot{z}}_{1}+k_{1}z_{1}=-m_{1}\ddot{y} & \left(z_{1}-z_{2}<\Delta x\right) \end{array}\right.$$

(2) $$HFGB:\;\left\{ \begin{array}{ll} m_{2}{\ddot{z}}_{2}+c_{2}{\dot{z}}_{2}+k_{2}z_{2}-k_{0}((z_{1}-z_{2})-\Delta x)=-m_{2}\ddot{y} & \left(z_{1}-z_{2}\geq \Delta x\right)\\ m_{2}{\ddot{z}}_{2}+c_{2}{\dot{z}}_{2}+k_{2}z_{2}=-m_{2}\ddot{y}\; & \left(z_{1}-z_{2}<\Delta x\right) \end{array}\right..$$

For simplifying the analysis, an assumption that beams and rope work on the linear range is adopted in the modeling. For harmonic base excitation *y(t) = Ysin(ωt)*, using 2*ζ*_1_*ω*_1_ *= c*_1_*/m*_1_, 2*ζ*_2_*ω*_2_ *= c*_2_*/m*_2_, *ω*_1_^2^ *= k*_1_*/m*_1_ and *ω*_2_^2^ *= k*_2_*/m*_2_, Equations (1) and (2) can be rewritten as follows:

(3) $$LFDB:{\ddot{z}}_{1}+2\zeta_{1}\omega_{1}{\dot{z}}_{1}+\omega_{1}{}^{2}z_{1}=\left\{ \begin{array}{ll} -\frac{k_{0}}{m_{1}}((z_{1}-z_{2})-\Delta x)+a\sin\omega t & \left(z_{1}-z_{2}\geq \Delta x\right)\\ a\sin\omega t & \left(z_{1}-z_{2}<\Delta x\right) \end{array}\right.$$

(4) $$HFGB:{\ddot{z}}_{2}+2\zeta_{2}\omega_{2}{\dot{z}}_{2}+\omega_{2}{}^{2}z_{2}=\left\{ \begin{array}{ll} +\frac{k_{0}}{m_{2}}((z_{1}-z_{2})-\Delta x)+a\sin\omega t & \left(z_{1}-z_{2}\geq \Delta x\right)\\ a\sin\omega t & \left(z_{1}-z_{2}<\Delta x\right) \end{array}\right.$$

where *ω*_1_ and *ζ*_1_ are the LFDB frequency and damping characteristics, *ω*_2_ and *ζ*_2_ are the HFGB frequency and damping characteristics, *a = Yω*^2^ is the acceleration amplitude of the base excitation. *m*_1_*_,_*_2_ = *m_b_/3 + m_p_/3 + m* [60], where *m_b_*, *m_p_* and *m* are the mass of substrate, piezoelectric layer, and proof mass. Thus, the displacements of the LFDB and HFGB versus the excitation frequency *ω* can be numerically derived based on Equations (3) and (4).

## 3. Experiment and Simulation Procedure

**Figure 4:**
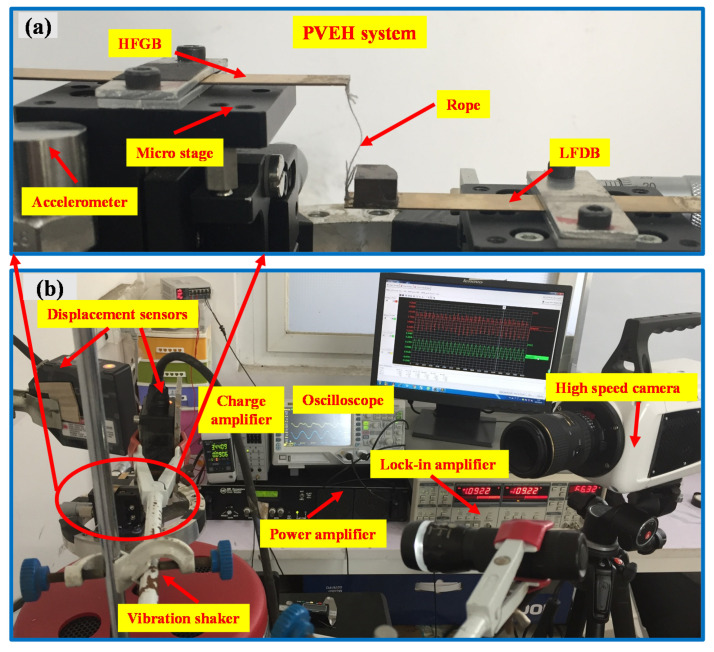
(**a**) Device fabrication and (**b**) detailed experimental setup of the PVEH system.

To determine the real-time displacement of LFDB and HFGB under different frequencies, a vibration monitoring system including a lock-in amplifier (STANFORD RESEARCH SYSTEM, Model SR830, Sunnyvale, CA, USA), vibration shaker (MB Dynamics, MODAL110, Cleveland, OH, USA), power amplifier (MB Dynamics, MB500VI, Cleveland, OH, USA), accelerometer (Baofei, JYD-2, Yangzhou, China), charge amplifier (Baofei, KD5002, Yangzhou, China), micro stages, two displacement sensors (KEYENCE, LK-G30/G10, Osaka, Japan) and a high-speed camera (PHANTOM, V611, Wayne, NJ, USA) were established, where the rope-driven PVEH system (see Figure 4) is fixed on a three-dimensional micro stage. In experiment, for the convenience of mechanical model vilification, LFDB and HFGB all use the pure beams without piezoelectric materials, and the detailed parameters are shown in Table 3. The damping ratios of LFDB and HFGB were measured from the exponentially decayed waveform using the relationship *ζ* = (1/2*nπ*)ln*A*_1_*/A_n_* [47], where *A*_1_ and *A_n_* are the amplitudes of peaks. The rope stiffness *k*_0_ is determined by the relationship *k*_0_ *= EA/L*, where *E* is young modulus measured by stress-strain relationship, *A* and *L* are cross-sectional area and length of rope determined by micrometer.

By adopting the parameters shown in Table 3, simulation results of LFDB and HFGB’s displacements can be achieved using MATLAB/SIMULINK^®^ model based on Equations (3) and (4). We firstly verify the feasibility of the mechanical model by comparing experimental results with simulation results. After that a parameter study was developed using the numerical simulation and experiments to understand the performance of the proposed PVEH. Specifically, we focus on the effect of rope, which is the easiest part for us to adjust once the beam structure is fabricated, in this paper.

## 4. Results and Discussion

**Figure 5:**
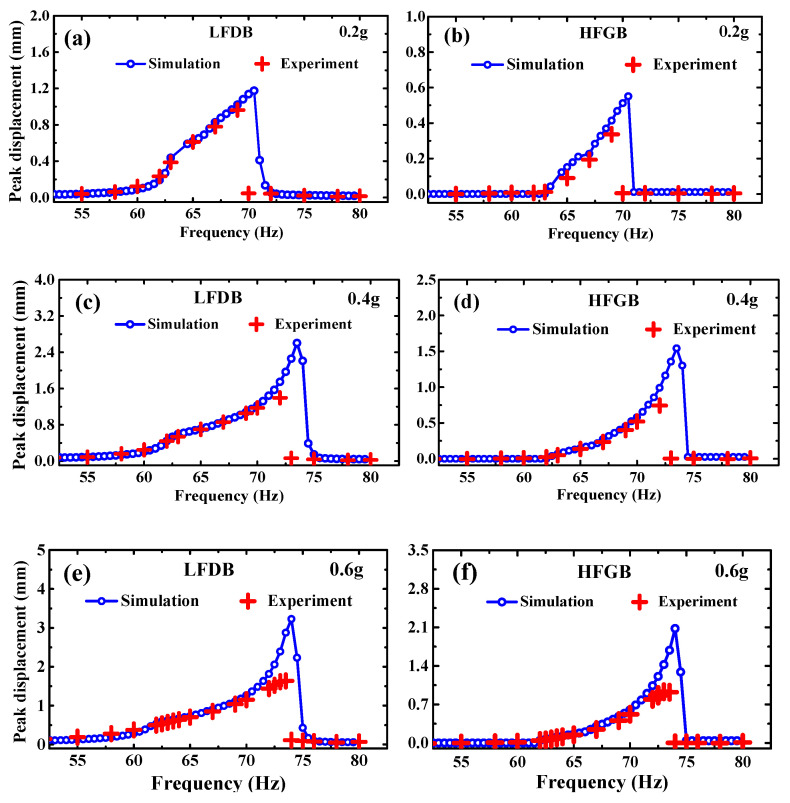
Experimental and simulation frequency responses of the peak displacement of LFDB with (**a**) *a =* 0.2 g, (**c**) *a =* 0.4 g and (**e**) *a =* 0.6 g; experimental and simulation frequency responses of the peak displacement of HFGB with (**b**) *a =* 0.2 g, (**d**) *a =* 0.4 g and (**f**) *a =* 0.6 g.

**Figure 6:**
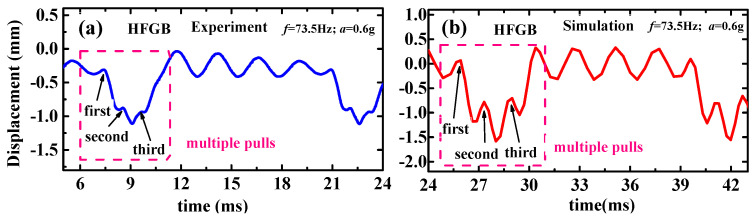
Typical real-time experimental (**a**) and simulation (**b**) displacement of HFGB.

**Figure 7:**
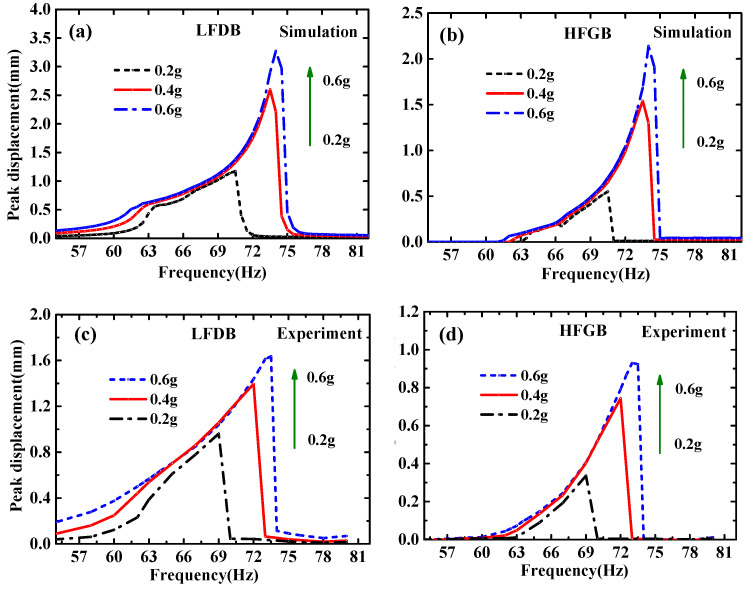
Frequency responses of the peak displacement of LFDB and HFGB for various accelerations under the condition of *Δx* = 0.5 mm and *k*_0_ = 1150 N/m: (**a**) simulation frequency responses of LFDB; (**b**) simulation frequency responses of HFGB; (**c**) experimental frequency responses of LFDB; (**d**) experimental frequency responses of HFGB.

**Figure 8:**
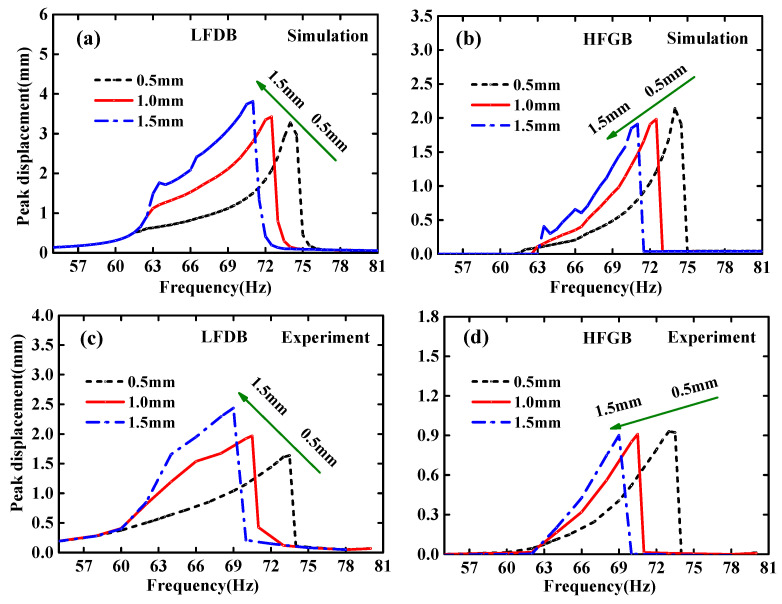
Frequency responses of the peak displacement of LFDB and HFGB for various rope-margins under the condition of *a =* 0.6 g and *k*_0_ = 1150 N/m: (**a**) simulation frequency responses of LFDB; (**b**) simulation frequency responses of HFGB; (**c**) experimental frequency responses of LFDB; (**d**) experimental frequency responses of HFGB.

Figure 5, Figure 6, Figure 7 and Figure 8 show the simulation and experimental results of the proposed PVEH for acceleration *a* from 0.2 g to 0.6 g, rope margin *Δx* from 0.5 to 1.5 mm and rope stiffness *k*_0_ from 170 to 2500 N/m. The peak displacement of LFDB and HFGB is used as output to evaluate the characteristics of the rope-driven PVEH under different frequencies, which is defined as the maximum displacement during the rope-driven phase where LFDB pulls HFGB and reaches the lowest place in the downward direction (shown in Figure 2).

As seen in these figures, the simulation results basically agree well with the experimental results, demonstrating the validity of our rope-driven PVEH modeling though there are slight mismatches in amplitude over special frequency range. Figure 5 shows typical simulation and experimental frequency responses curves of LFDB and HFGB with *Δx* = 0.5 mm, *k*_0_ = 1150 N/m, and *a* = 0.2 g, 0.4 g, 0.6 g. It can be seen that the simulation curve coincide the experimental curve in most frequency ranges, and the minimum error of displacement can reach 1.3%. However, the displacement discrepancy of simulation and experiment is more obvious when excitation frequencies get close to the end-frequency point (corresponding to the frequency point where the output suddenly drops to zero). Interestingly, real-time experimental and simulation displacement curves of HFGB (shown in Figure 6) demonstrated that shape of dynamic motion curves are still kept similar and has same pulling times. The mismatches in amplitude over the end-frequency range are probably due to two reasons. Firstly, the closer to the end-frequency point, the more strongly the whole PVEH system vibrates, which makes the beams and rope work on the nonlinear range possibly. However, the mechanical model we built gives no consideration to the nonlinear terms of beam movements. Furthermore, it can be seen that the higher acceleration is, the mismatch over the end-frequency range is more obvious by comparing Figure 5a,b with Figure 5c–f. Secondly, the rope is equivalent to a spring without damper in the established model, which may be oversimplified, considering the complicate movement of HFGB and LFDB in the multiple-pulling stage (shown in Figure 6). Hence, the non-linear terms of beams and the good equivalent of rope under strong excitation should be addressed for the model improvement in the future.

In general, the model established in this paper can be used to predict the characteristics of the rope-driven PVEH effectively. For example, in Figure 7, both simulation and experimental results show that wider bandwidth and higher output displacement of LFDB and HFGB can be observed when the excitation increases from 0.2 g to 0.6 g. Using the numerical simulation system in this paper, the characteristic of PVEH can be predicted qualitatively before fabricating the device for guiding the design of the PVEH.

According to the established mechanical model, we know that the performance of the PVEH is affected by multiple parameters, which mainly includes geometric parameters of LFDB and HFGB, margin and stiffness of rope, and excitation acceleration level. As the PVEHs applied in a specific application, once the PVEHs are fabricated, the characteristics of PVEHs will be fixed. Usually, owing to the fabrication error, there would be a different performance between the designed PVEHs and the fabricated PVEHs. If to change the performance of PVEHs, it will be difficult and inconvenient by changing the parameters of LFDB or HFGB, especially for the MEMS PVEHs, unless the devices are re-fabricated, while by adding the rope to PVEHs, only controlling the rope margin, the desired performance can be realized effectively. Hence, we mainly focus on the parameter study of rope margin and stiffness by using numerical simulation and experimental investigation.

Figure 8 shows the frequency responses of the peak displacement of LFDB and HFGB for different rope-margins. It can be seen from the simulation and experimental results that the performance of PVEH can be tuned by controlling the rope length. Taking the frequency responses curves in Figure 8b of HFGB as an example, as the rope margin changing from 1.5 mm to 0.5 mm, the bandwidth can be broadened from 7.5 Hz (63.5–71 Hz) to 12.5 Hz (62–74.5 Hz), although there is a slight drop in the output near the end-frequency point. Thus, changing rope could become a more convenient choice for adjusting the performance of the PVEHs applied in the real applications, and can also increase the tolerance of fabrication.

**Figure 9:**
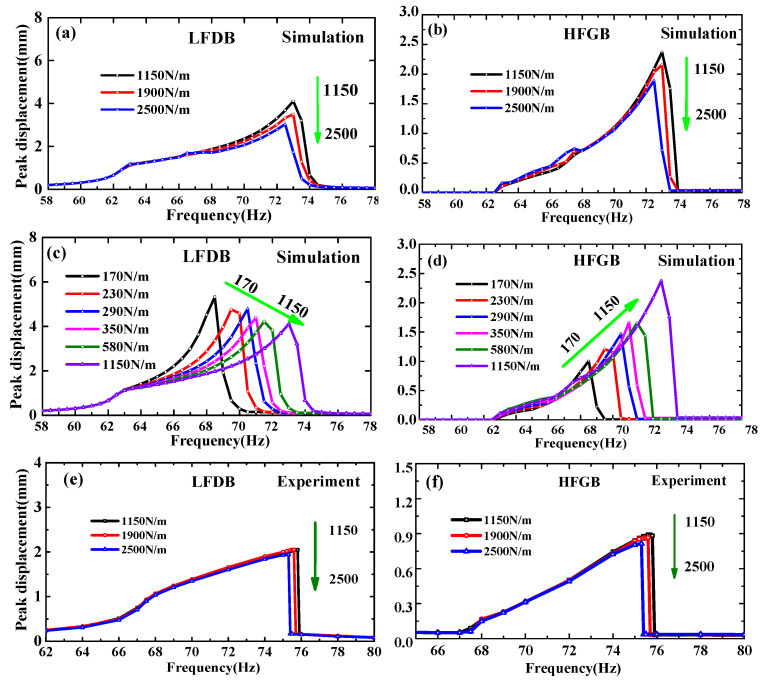
Frequency responses of the peak displacement of LFDB and HFGB for various rope stiffness under the condition of *a =* 0.6 g and *Δx =* 1.0 mm: (**a**) simulation frequency responses of LFDB under *k*_0_ = 1150–2500 N/m; (**b**) simulation frequency responses of HFGB under *k*_0_ = 1150–2500 N/m; (**c**) simulation frequency responses of LFDB under *k*_0_ = 170–1150 N/m; (**d**) simulation frequency responses of HFGB under *k*_0_ = 170–1150 N/m; (**e**) experimental frequency responses of LFDB under *k*_0_ = 1150–2500 N/m; (**f**) experimental frequency responses of HFGB under *k*_0_ = 1150–2500 N/m.

Figure 9 depicts frequency responses of the peak displacement of LFDB and HFGB for various rope stiffness *k*_0_ changing from 1150 to 2500 N/m. Both simulation and experiments suggest that bandwidth and output displacement of LFDB and HFGB (shown in Figure 9a,b,e,f) are weakened but not much compared to the effect of rope margin when increasing the rope stiffness such as from 1150 to 2500 N/m. To further understanding the effect of rope margin, small rope stiffness less than 1150 N/m was investigated by simulation, which is shown in Figure 9c,d. In contrast to the conclusions as rope stiffness *k*_0_ changing from 1150 to 2500 N/m, the bandwidth of LFDB increases when the rope stiffness is at a range of 170–1150 N/m, whereas its displacement decreases (see in Figure 9c). As for HFGB, the bandwidth increases, whereas the displacement hardly decreases (see in Figure 9d). It means that the bandwidth and output of rope-driven PVEH can also be adjusted by rope stiffness, and could be improved further if the stiffness of rope increases in a certain range. Overall, for practical applications, adjusting rope margin is a better and more effective way to change the rope-driven PVEH’s performance to achieve tunable energy harvesting.

## 5. Conclusions

In conclusion, a mechanical mass-spring-damper model of the rope-driven PVEH with one LFDB connecting one HFGB by a rope was built using lumped parameter modeling approach, and the dynamic equations for predicting the dynamic movement of LFDB and HFGB were established, respectively. Based on the equations using MATLAB/SIMULINK^®^ model, the effects of multiple parameters including excitation acceleration, rope margin and stiffness were investigated by numerical simulation and experiments. Overall, the results show that the established model in this study can be used to predict the behaviors of our rope-driven PVEH though there are some mismatches near the end-frequency point, which can provide guidance for the design of the rope-driven PVEH before fabricating it. The parameter study suggests that rope-driven PVEH’s performance can be optimized by multiple parameters. Adjusting rope is the most convenient choice for optimizing the performance of the PVEH in variable environments without refabricating the device, this tunable performance of the proposed rope-driven PVEH system makes it promising for vibration energy harvesting in wideband environments with low frequency.

## Figures and Tables

**Table 1:** Typical micro piezoelectric vibration energy harvesters (PVEHs).

References	Effective Volume (mm^3^)	Power (µW)	Acceleration (g)	Frequency (Hz)
Jeon et al. [22]	0.027	1.01	10.8	13,900
Renaud et al. [23]	1.845	40	1.9	1800
Shen et al. [24]	0.6520	2.15	2.0	462.5
Muralt et al. [25]	0.48	1.4	2.0	870
Elfrink et al. [26]	15	69	0.2	599
Park et al. [27]	1.05	1.1	0.39	528
Fang et al. [28]	0.78	2.16	1.0	608
Kanno.et al. [29]	0.168	1.1	1.0	1036

**Table 2:** Common vibration sources [30,31].

Vibration Sources	Acceleration (g)	Frequency (Hz)
Vanitation pipe	0.02–0.15	60
Lathe	1.0	70
Truck/Car engine	0.052–0.198	37
Human walking	0.2–0.3	2–3
Car instrument panel	0.3	13
Three-axis machine	1.0	70
Office building near the road	0.02–0.15	60–100
Tunnel train secondary vibration	0.0026	15–25

**Table 3:** Simulation and experiment parameters of LFDB, HFGB and the rope.

Parameters	LFDB	HFGB	Rope
Length (mm)	24.32	19.51	18.7
Width (mm)	6.00	6.00	-
Thickness (mm)	0.28	0.28	-
Diameter (mm)	-	-	0.1
Proof mass (g)	1.93	0.00	-
Frequency (Hz)	63.7	410.3	-
Young modulus (Gpa)	90	90	2.7
Density(kg/m^3^)	8800	8800	-
Damping ratio	2.56 × 10^−3^	5.79 × 10^−3^	-
